# ‘A Completely Different Person’: Embodied Dialectics and Biographical Disruption After Stroke

**DOI:** 10.1111/1467-9566.70113

**Published:** 2025-11-06

**Authors:** Sophie Rowland‐Coomber, Eleanor Stevens, Christopher McKevitt, Nicholas J. Williamson, Iman Muzafar, Timothy Neate, Martin Chapman, Ajay Bhalla, Charles D. A. Wolfe, Iain J. Marshall, David Wyatt

**Affiliations:** ^1^ Department of Population Health Sciences King's College London London UK; ^2^ Department of Language and Communication Science School of Health & Psychological Sciences City St George's, University of London London UK; ^3^ Department of Informatics King's College London London UK; ^4^ Ageing Health and Stroke Guy's and St Thomas' National Health Service Foundation Trust London UK; ^5^ NIHR Applied Research Collaboration (ARC) South London at King's College Hospital NHS Foundation Trust London UK

**Keywords:** biographical disruption, embodied dialectics, informal care, stroke

## Abstract

Stroke is a leading cause of complex disability, with many survivors experiencing mobility, cognitive and/or speech and language impairment. This paper explores the relationship between biographical disruption and body studies through experiences of informal care in stroke. Drawing on narratives from 41 interviews with stroke survivors and their wider support network, we use Michael Bury's concept of ‘biographical disruption’ alongside body studies theorists to construct a framework to understand the role of embodiment within biographical disruption. We draw on Victoria Cluley and colleagues' concept of ‘biographical dialectics’ to reveal, through our data, an ‘embodied dialectics’, where past and present embodied experiences of chronic illness exist in a productive tension. We identify three distinct but interlinking aspects: (i) contradictions between past, present and future embodied understandings are generative, leading individuals to produce new forms of embodied knowledge; (ii) tensions create motion, ensuring ongoing dialectical processes that generate creative adaptations and conversations in relation to informal care and embodied practices post‐stroke and (iii) these processes are ongoing as the competing demands of autonomy and dependence continue to generate new challenges. In doing so, we highlight the roles of socio‐cultural practices and expectations in shaping individual and collective embodied understandings of illness and subsequent disruption.

## 1 Introduction

Each year, 15 million individuals experience stroke, with 5 million of those left permanently disabled (World Health Organisation 2025). Stroke impacts mobility, cognition, vision, speech, language and the social world in which individuals operate (Kristo and Mowll 2022; Morris et al. 2017), whilst also increasing psychological and emotional needs (Harrison et al. 2017), with some individuals experiencing long‐term depression (Liu et al. 2025). Although the psychological impact of stroke may not be as apparent as the physical, stroke does shape how individuals experience independence and identity (Mavaddat et al. 2021).

Body studies emerged as a compelling field in which to narrate the principal effects of stroke, with scholars considering the embodied impact (Lo et al. 2023). Rehabilitation can prove a challenging process for stroke survivors as they come to terms with their body's abilities and the disruption which the stroke has caused not only to their everyday lives but sense of self (Arntzen et al. 2015), with the body serving as a ‘narrative resource’ as they create ‘ongoing identity’ post‐stroke (Faircloth et al. 2004, 244). Timothy et al. (2016, 1573) have described the relationship between body and self after stroke as in a state of ‘flux between divergence and cohesion’, whilst Stott et al. (2021) refer to stroke survivors' uncertainty in their bodies post‐stroke versus pre‐stroke. Collectively, these studies reveal that stroke recovery is fundamentally an embodied process of identity reconstruction, where rehabilitation must address not just the physical function but the complex negotiation between disrupted bodily experience and evolving sense of self.

The disruptive effect of stroke has been described as a ‘biographical attack’ in which the body seeks stability as a ‘legitimate entity’ (Faircloth et al. 2004, 257, Finch et al. 2021). This builds on previous scholarship in which chronic illness was viewed as an ‘assault’ on both the person's ‘physical health’ and ‘sense of identity’ thus having crucial implications for individuals' social responses and interactions (Bury 1991). Central to Bury's argument was to establish a critical avenue of discussion regarding the relationship between health, sense of self and role within society (Bury 1982; Williams 2000). Engman (2019) examined the relationship between biographical disruption and embodiment to consider further how we might view experiences of illness. Cluley et al. (2023), in considering the use of haemodialysis by people with end‐stage kidney disease, developed the term ‘biographical dialectics’ to capture the dissatisfaction and ongoing disruption that occurs because of chronic illness. Existing applications have predominantly focused on individual experiences of illness, with limited attention to how these biographical and embodied negotiations unfold within relational contexts of care, specifically. This leaves underexplored how the ongoing, interactional nature of informal care relationships might reveal new dimensions of biographical disruption and embodiment over time.

In this paper, we advance the conception of biographical disruption and its relationship to embodiment by considering experiences of stroke over time, within the context of informal care. Through consideration of individuals' changing and re‐negotiated bodies, identities over time are recreated and re‐imagined. In recognising the work of Cluley et al. (2023), we aim to provide further synthesis of ‘biographical dialectics’ and body studies. Through suggesting an ‘embodied dialectics’, we recognise the body as an active site of contradiction where past and present embodied experiences exist in productive tension.

### 1.1 Biographical Disruption Embodied

Biographical disruption has fulfiled a critical role in narrating experiences of chronic illness (Locock and Ziebland 2015). Because the concept's development, several scholars have noted the importance of contextualisation and demographic data when considering the usefulness of the concept in narrating experiences of chronic illness (Wedgwood et al. 2020; Cluley et al. 2021; Engman 2019; Williams 2020). Yet, despite this, this concept still has an essential role to play when attempting to understand experiences of stroke, and what body studies might add to our understanding of these discussions surrounding disruption. Bury (1982) identified three key forms to examine chronic illness: ‘self‐identity’/‘self‐consciousness’; ‘normalisation’ and the ‘social and material’ impact. Thus, identity is an active creation in which the self is reconstructed and revised to understand experiences, such as stroke (Holstein and Gubrium 2000).

Faircloth et al. (2004) suggest that ‘biographical flow’ is more accurate than ‘disruption’ to describe the continued dialogue between self, body and identity, with many stroke survivors experiencing an altered and ‘unfamiliar’ self‐image (Nasr et al. 2016, 4). Cluley et al. (2023, 9) attempt to capture the ‘tension’ that exists amongst individuals with chronic illness between ‘current position’ and ‘desired end point’. Through the concept of ‘biographical dialectics’, biographical disruption is shown to be ‘fluid’, ‘relational’ and to ‘create further disruption’ (Cluley et al. 2023, 8). Stroke often causes cognitive impairments and communication difficulties alongside physical limitations, and these various impacts are brought into focus depending on the context and time point post‐stroke, making changes in identity difficult to summarise and articulate.

In examining the ‘normal’ within disruption, Bury (1982) considered the impact of illness in removing physical capabilities which individuals had previously taken for granted. Leder (1990) examines the relationship between the body and illness, referring to ‘social dys‐appearance’, in which the body may emerge as an ‘alien’, ‘prison’ or ‘tomb’ in which an individual is ‘trapped’, searching for the normal. This understanding of the body was devised from the theoretical discussions of Merleau‐Ponty (1964), who described the body as a ‘mechanism’ to describe the body consciousness which exists beyond the active components of the body schema. Engman (2019) draws on this theoretical discussion to present an ‘embodied orientation’ in which illness does not create disruption but rather affects one's bodily ability to engage in daily life, thus fostering presence and/or absence (Engman 2019, 126). Stroke, in altering the ‘normal’, can lead to often ‘unintelligible’ and ‘disturbing changes’, leaving survivors feeling that their body was unpredictable, demanding tools or remedies to assist and incorporate into their identity (Kvinge and Kirkvold 2003).

Stroke survivors' bodies can feel ‘distant’, ‘conflicted’ between current and previous bodily understanding (Norris et al. 2010). In ‘biographical dialectics’, Cluley et al. (2023) suggest a similar cycle of disruption, in which solving one problem (thesis) can lead to another problem (antithesis) that needs addressing (synthesis). In the stroke survivor, the body that was (pre‐stroke capabilities) informs the body that is (current limited body) and can inform the body that might be (hoped for recovery/fear of decline). Thus, within this ‘embodied dialectic’ and engaging with Faircloth et al. (2004), it is evident that factors such as age, pre‐existing health conditions and previous experience of stroke prove important in enabling individuals to come to terms with the realities of their health and subsequent bodily limitations.

Lastly, Bury (1982) considered the ‘social and material’ impact of illness (social, financial and health resources). Bourdieu's Outline of a *Theory of Practice* (1977) had previously explored the social complexities of human interaction in individuals' navigation of the body and illness. For Bourdieu, it was important to consider the interplay between an individual's habitus and capital, as social status and resources were important in determining individuals' narration of experience. Sadler et al. (2014) applies Bourdieu's concept of capital to explore issues of access to stroke care, demonstrating how some individuals are better equipped to manage after stroke because of the varying existing resources (capitals), including the wider networks of support they have access to. Sadler et al. (2014) prompts us to recognise the importance of community in life after stroke, and specifically in this article to consider the relationship between stroke and embodiment as a collective as well as individual experience.

The role of individual identity in informing and formulating a collective identity is discussed by Hydén and Forsblad (2020) in the context of dementia and memory, in which they argue identities are ‘constructed interactively’ through the environment that surrounds them. In informal care practices we gain insight into the social, economic and personal interactions that shape a stroke survivor's identity and embodied understanding. The role of carers has been recognised by scholars as crucial in a stroke survivor's negotiation of identity with chronic illness. As chronic illness alters relationships and gender roles (Quinn et al. 2014; Van Dongen et al. 2014), it generates a grieving process for the life that is altered or lost by both stroke survivor and carer (Olivier et al. 2018; Lu et al. 2019), alongside the additional social and economic burdens that individuals face as a result of their caring responsibilities (Broese van Groenou and De Boer 2016).

This view of identity as a ‘collaborative process’ is developed further in Hydén’s (2021) writing on dementia. Here, identity is an illustration of an individual's connection to their social environment. Moreover, individuals may choose to restructure ‘cognitive’ and ‘semiotic’ resources to communicate their identity and the identity of others who care for them. Informal carers are often at the forefront of the recovery process: in our final theme, collective embodiment, the research draws on carers' accounts alongside stroke survivors', to articulate what Stott et al. (2021, 1351) describe as the ‘disappearing body’, referring to the body's alterations following a stroke.

Having outlined key concepts within both biographical disruption and body studies, we have explored how this conceptualisation might add to an understanding of the long‐term effects of stroke. We examined Bury's three key framings of biographical disruption whilst integrating this with relevant body theories. Through our discussion of stroke, embodiment and disruption, we draw on Cluley et al. (2023) concept of ‘biographical dialectics’ to present an ‘embodied dialectics’ that aims to articulate the ongoing productive tensions between multiple embodied states that generate continuous cycles of bodily awareness, adaptation and new forms of bodily knowledge. This paper focuses on the experience of informal care within stroke. This enables us to consider the extent to which individuals are forced to change and negotiate their body and identity as a stroke survivor, and the impact this imposes on their wider network of support. Through these social and cultural interactions and interview discussions, the stroke body is re‐examined within multiple contexts and at different points in time to reveal an individual and collective understanding of disruption.

## 2 Methods

In this study, 41 participants were included (via telephone [*N* = 39] or face‐to‐face [*N* = 2]). Where possible, interviews were completed with stroke survivors (*N* = 27). In cases where individuals felt unable to participate in interviews because of stroke impairments (such as communication difficulties, e.g. aphasia), we spoke to a member of their wider support network made up of relatives, friends and carers (*N* = 14). Participants comprised male (*N* = 30) and female (*N* = 11) individuals aged 23–93 years, whose stroke had occurred between 2007–2022.

We recruited participants through the South London Stroke Register (SLSR), an ongoing observational study monitoring the incidence and outcomes of stroke. Geographically restricted to the South London (UK) area, the study follows up individuals from 3 months through to 15 years following their first stroke (Marshall et al. 2023). We took care in the construction of interview questions to address our overall research aims but also ease participants into discussions of more sensitive topics relating to their informal care practices, ensuring participants felt confident in discussing both lived experiences and realities of informal care (McKevitt 2000). We consulted our national stroke Patient and Public Involvement (PPI) group both in the construction of interview questions and in the interpretation of results. We asked participants questions relating to activities of daily living, medication and socialisation alongside how the SLSR follow‐up interviews provided them with a space to discuss their experiences of informal care and how this could be enhanced. Ethical approval for the study was obtained through The Health Research Authority (REC Reference 21/WS/0145).

Much of the research undertaken on the relationship between disruption and stroke has focused on experiences within the first year of stroke, as the period in which the greatest impacts are felt (Pound et al. 1998; Faircloth et al. 2004). Narratives of family, friends and carers served to offer a more holistic understanding of the impact of stroke on the sense of self and an individual's relationship with their health, as they were often essential in negotiating informal care (see Broese van Groenou and De Boer 2016). Stroke severity varied with participants revealing impacts to their health such as complications with speech, fatigue, reduced mobility and mental health problems.

The analysis was conducted thematically, with the aim to create a space in which to critically reflect and unite participant narratives of lived realities, altered perspectives and experiences (Braun and Clarke 2022). Coding was operationalised using NVivo. During this first screening of transcripts, authors (NW), (IM) and (SRC) noted practices of informal care with particular attention as to how these were understood and implemented by stroke survivors and carers. As part of this, changes to the physical body were highlighted as important in narrating stroke survivors' and carers' experiences of informal care. In a second screening, author (SRC) focused on how bodily changes might impact their notion of identity, thus shaping individuals' understanding of their sense of ‘normal’ and ‘self’ within their social and cultural environment alongside stroke survivor/carer relationships. The topic of embodiment seemed relevant to participant discussion within the interviews, and was discussed with author (DW), before wider analysis of all interviews was undertaken by all three authors. Over several meetings with these authors and author (DW), codes were refined and themes were identified in relation to informal care, rendering into focus embodiment as a key theme in which to narrate long‐term experiences of informal care. In the following sections, participants are referred to using pseudonyms.

## 3 Results

Through discussion of their experiences of informal care, these narratives from stroke survivors and carers illustrate stroke as ‘embodied dialectics’ that impacts the construction of their past, present and future identities. Participants' discussion of identity focused firstly on the unfamiliarity that they experienced in relation to their body following their stroke, and the sense of unease and mistrust that this created in finding the ‘normal’ (or rather adjusting to the bodily changes), in performing everyday household and social tasks. Secondly, participants identified the disruption that their stroke had caused to their bodies with varying responses, with many having to rethink the social and physical environments within which they operated. In exploring the experiences of stroke survivors, it also became apparent that the disruptive effects of stroke were felt not only by the survivor but their wider network of support too, as they were forced to navigate the consequences of these bodily changes with social and economic consequences.

### 3.1 Identifying the ‘Normal’ in an Unreliable Post‐Stroke *Body*

Stroke diminished individuals' confidence in their health and subsequently their bodies' capabilities. This is evident in the account of Mateo, who presents evidence of bodily ‘dys‐appearance’ (Leder 1990) as he struggled to come to terms with the decreased ability of his body post‐stroke:

I didn’t kind of imagine myself like not being able to do something that I want to do. That will affect me … I always felt very much in charge of what was happening … And then, suddenly, it’s like I’m not actually, I’m not in charge of anything at all, I need some help from someone else. And that is really hard to actually, yes, to understand and to accept.

(Mateo)

Acceptance of the limitations that his stroke placed upon his body proved disruptive for Mateo, challenging his sense of autonomy. The recognition of ‘the need for help’ proved significant, in this case highlighting the disconnect between stroke survivors' expectations of physical recovery and the reality of their health. For many individuals, their bodies appeared unrecognisable following a stroke, and therefore part of adapting to life post‐stroke included adjustment to new bodily constraints:

I’m kind of the complete opposite to what I was… you know, walking and doing the things quickly. Now I – if I’m going out for a walk, I usually have to – I have a walking stick because it’s, it’s hard – it’s, I know I can still walk but it’s getting tired, or I have to use the stick. I only use the walking stick for moral support because it’s – I found myself, you know, going over on the left side or sort of becoming hunched over. You know that sort of thing, it’s just making me more, more aware of what I can’t do what I used to do, when I had loads of energy. And now, I can’t.

(Richard)

This account from Richard reflects the transformation of the stroke survivors' body with an emphasis on physical limitations. Richard, alongside other participants, emphasised the effects that the stroke had had on his physical capabilities, describing himself as the ‘complete opposite to what I was’. Despite efforts to maintain previously normal components of daily life, the use of walking aids served as a physical reminder to the individuals of their stroke and encapsulated the evident loss of confidence in their body's physical abilities. More notably, through Richard we see the ‘embodied dialectic’ unfold as he remembers his body's previous capabilities in terms of what he ‘was able to do’—walking quickly or having energy. His current physical limitations and new sensations create an ‘antithesis’, as Richard describes his need for a walking stick and tendency to hunch. Lastly, we see how the need to find solutions creates new forms of embodiment as Richard's walking stick offers ‘moral support’ but also embodied awareness of dependence.

Aarin described the loss of sport from his life as ‘traumatic’. The intense level of ‘pain’ and ‘aches’ made it impossible for him to engage in the sporting activities that he had enjoyed prior to his stroke:

I feel down so many times, cry for no reason, just wandering off thinking about what will happen next. It’s just very traumatic it’s been since. I know people go through a lot as well. I haven’t been able to do any sport now with my pain and aches and my right side is just very painful sometimes – well, sometimes, I say sometimes, it’s every day.

(Aarin)

Within Aarin's account we see how he retains a passion for sports he can no longer play due to his bodily changes. For a stroke survivor, the sense of recovery was associated with the ability to maintain activities enjoyed and undertaken prior to their stroke. This, in turn, brought attention to the body, which forced individuals to reflect on their physical capabilities (Eilertsen et al. 2010). For Aarin, his inability to play sports again acted as a reminder that he could no longer feel assured of his body. The stroke, an event rupturing his sense of self and bodily autonomy, had left him concerned about ‘what will happen next’. In their recovery, stroke survivors grappled with the relationship between their health and emotions, and the need to regain trust in their body's capabilities. Mistrust in their body post‐stroke was discussed by Linda:

I’m still not able to live on my own and go out on my own because I’m, you know, I’m frightened, that you know, something might happen when I was on my own.

(Linda)

For Linda, ‘fear’ that the stroke might recur meant she had been forced to relinquish her independence and was ‘unable to live on her own’. She sought to overcome the unreliability of her body through the support of those around her. Independence, for several participants, proved intrinsically linked to their health and trust in their bodies to continue to lead the same life that they had had prior to their stroke. For most, this was not possible. Instead, trust in the body needed to be built over time. Recovery for stroke survivors was about learning to accept the current state of their bodies and understanding and adjusting to these capabilities.

This search for ‘normalcy’, or return to activities undertaken prior to stroke, proved a focus for individuals as they sought to regain control of their bodies, suppressing the ‘social dys‐appearance' or sense of otherness that emerges within individuals as they seek to regain an understanding of their bodies within their social world (Leder 1990). Therefore, it is not surprising that for many participants stroke manifested as not just a medical condition but a ‘biographical attack’, the disruptive effects of which needed to be minimised to maintain the body's ‘stability’. Stroke survivors were clearly conscious of but uneasy about their body's capabilities; this is something that shaped their social experiences and relationships. They could no longer trust in their health; their bodies had let them down and, as a result, they were left vulnerable.

### 3.2 Emboldened Identities: The Social Construction of the Post‐Stroke Body

The disruption that individuals had experienced because of their stroke, as highlighted through social and informal care interactions, fostered an intense self‐awareness of their bodies. Narratives from stroke survivors reveal a sense of frustration: unable to control public projections of their bodily appearance as they once had, they felt exposed within the social situations of their everyday lives. Kevin described the extreme ‘self‐consciousness’ following his stroke, in part linked to the deep awareness that he had of his bodily image:

It’s the weight of carrying it and like, as I said to you previously, where I feel so tired … I’m very self‐conscious now because it’s not a nice world we’re living in at the moment … And I’m very self‐conscious of being out, when I’m around a lot of people.

(Kevin)

The changes that his body had undergone, and the current physical limitations of his body, left Kevin fatigued and self‐conscious in public places. His awareness of the alterations that his body had undergone because of his stroke fostered a sense of uneasiness about how wider society might choose to interact with or respond to him. The disrupting effect of stroke upon individuals' self‐consciousness was felt not only within activities of daily life but also in navigating social relationships post‐stroke.

Stroke survivors often found it difficult to articulate their resentment and dismay about the situation in which they now found themselves. Humour, as an expression of mood as well as mindset. allowed individuals to confront and endure the physical implications of their stroke, as well as enabling relief and support for their wider support network (Stott et al. 2021, 1353). The inability to communicate easily proved frustrating for participants. Speech difficulties, such as aphasia, commonly affect stroke survivors, with many experiencing these problems consequently having depression and anxiety (Kristo and Mowll 2022; Shehata et al. 2015). As a result of his stroke, Edmund had aphasia, which meant he often struggled to pronounce his partner's name. During his interview, Edmund outlined the routine he and his partner would perform to alleviate the awkwardness of the situation:

I used to say [partner’s name], my partner, ‘Val.’ And she said, ‘No, I’m [partner’s name].’ ‘Oh, hang on, go and get the pegs.’ So, I used to put pegs on, and they used to take the mickey out of me. I am getting better; I am getting better. I’m able to talk pretty reasonably. And since then, I’ve been fine.

(Edmund)

In placing the ‘pegs on’ (clothes pegs clipped on his lips to support him to make specific sounds), Edmund gained a sense of ownership over the physical limitations of his body and transformed his speech therapy into a shared and ongoing source of humour with his wider network of support. The nature and use of humour in stroke recovery varied, serving to overcome ‘tribulations and ensure a sense of ‘normalcy’ (Torregosa et al. 2018, 364). Others have described humour as a mechanism by which stroke survivors seek to ‘evade the emotional discomfort’; an expression of ‘embarrassment’ or ‘weirdness’ that may prove too difficult to communicate otherwise (Stott et al. 2021, 1353). More specifically, Sherratt and Simmons‐Mackie (2015) have highlighted the use of humour within aphasia groups, helping to ensure individuals feel included and enabling them to form social support networks. In contrast, Adam found humour used by friends and family around his speech was detrimental to his recovery, in that it made him more conscious of the impact that the stroke had had upon his body. Adam described how aphasia had severely restricted his ability to communicate confidently:

It’s really, really hard, just like to put a word together, but if I don’t concentrate, I would just mess up. Just like I can’t have my friends anymore around me because when they are talking, when they talk, I don’t even know what they are talking about … They are making fun of me, and that has affected me emotionally.

(Adam)

As Adam notes, the stroke had left him isolated, as he no longer felt able to maintain the same level of connection that he had once had with his friends due to his communication difficulties. Acts of ‘making fun’, such as those displayed by his friendship group, proved emotionally draining and left him feeling a level of self‐consciousness such that he found it easier to cease contact with them. Such displacements highlighted the profundity of the effects that the stroke had had on Adam's body: his condition not only changed relationships but ‘transformed identities’ and ‘(changed) roles in social context’ (Nasr et al. 2016). The increased levels of self‐consciousness that stroke survivors experienced had implications for not only how they saw their relationship with wider society but also for their wider support network, who were often at the forefront of addressing the outcomes of life post‐stroke.

During interviews, concerns were voiced within wider support networks as to the impact that the physical disruption had on stroke survivors' bodily consciousness, and the potential effects of emotional strain on recovery post‐stroke. Brian articulated the impact that his relative's stroke had had, the awareness of bodily changes fostering a heightened sense of self‐image, which were often confounded by feelings of loneliness and isolation from friends and relatives:

He’s, he’s lost the confidence about the way he looks, I think that’s another issue. Even though he is complaining about family not visiting him. He still has this awareness that he doesn’t want that many people to come and see him because of the way he looks, you know. And one of the things he will say to me that I should bring all his old photographs so people can see what he used to look like when he was young‐ when he was a young man.

(Brian, cousin of Joseph)

In asking Brian to ‘bring his old photographs’, Joseph attempted to take control of his identity and how he is perceived—the photos are his tool by which he controls his own life narrative. These attempts to control an identity narrative by Joseph, however, also reinforce temporal body rupture. He is not just the person here and now who has had a stroke but he was previously young, youthful and able‐bodied. Neate et al. (2022) noted similar observations when considering how people with aphasia presented themselves in videoconference (as opposed to in person at a charity centre), with individuals drawing on their environment and belongings.

Whilst accepting that stroke impacts the physical body, individuals face challenges in learning to adapt and accept that these changes might impact how others perceive them. One stroke survivor discussed the changes to their vocal cords resulting from their stroke. Having performed with several prestigious orchestras since the early 2000s, the participant felt they were unable to rely on their voice, as the quality had deteriorated. It was evident that this had proved regrettable for the participant, as the stroke had disrupted their body:

I’m a bass, a lower bass. The very top of my voice disappeared, the sort of baritone type bits above middle C, they became very unpredictable, shall we say, if not impossible. And the lower end, I would say, just dropped a little bit. But I’ve always been able to go quite deep. So, that was something which was annoying. And my voice partly recovered but it’s never quite been the same as before the stroke.

(William)

Changes to voice, particularly when intertwined with their work and professional identity as a singer, appeared frustrating and arduous to overcome. The ability to complete tasks as before, as exemplified in social situations such as work, appeared particularly difficult to address, as in the case of William's body where memory of vocal range was evident but unavailable. The managing of the body's capabilities to perform tasks proved important to both physical and mental recovery, in learning to understand bodily limitations. Recognition of changes in abilities evoked negative emotions, strongly connected with their sense of self‐image as displayed in social activities such as working life. This is evident in the account of a participant who had been forced to give up work because of their stroke:

I was a hard worker before. This thing has slowed me down, it’s slowed me down. And like now, I find myself a useless person. You know, that, that it’s so hard to say that, that I’m a useless person. Because, for me, I’m useless because I can’t provide for my family. I can’t provide for myself.

(Adam)

In denying Adam the ability to work, the stroke had deprived him of his ‘usefulness’, fostering negative feelings towards self‐image and thus resulting in low self‐esteem. The ‘loss of role and status in society’ (Mavaddat et al. 2021, 7) as seen in Adam's case further accentuates the changes that their body underwent. Other interview participants sought to highlight the overwhelming nature of their stroke stating, ‘I feel down so many times, cry for no reason’, with another participant noting that they ‘feel a bit depressed’. Many stroke survivors experienced a sense of isolation from themselves as well as from their family and friends, with many individuals struggling to find a connection to their sense of self prior to their stroke.

The changing physicality of stroke suggests a ‘re‐route’ in communication for people with aphasia and other cognitive stroke side effects—what cannot be said with voice, must be said with the assistance of hands or objects, as illustrated by Edmund who uses pegs to aid his speech. Therefore, it is not surprising that for several of our participants, stroke fostered an intense self‐awareness of the body and, more importantly, the limits of an individual's capabilities. Whereas participants were not specifically asked to document their bodily capabilities prior to stroke, it was clear that the stroke had left them with a sense of ‘estrangement’ towards themselves (see Murray and Harrison 2004). Work, as a form of identity, proved for stroke survivors to be an area in which the impact of this was most keenly felt, in terms of measuring levels of both physical and mental recovery post‐stroke. As a result of the stroke, many participants were forced to negotiate the new limitations of their bodies, the impact of which was reflected in individuals' social interactions and perceived self‐image.

### 3.3 Collective Identity: Changed Relationships Between Stroke Survivors and Carers

Embodiment, whilst primarily an individualised experience, can also be viewed as collective. Shilling (2017) described this as ‘body pedagogics’—recognising the role that both social and cultural practices and experiences play in dictating bodily consciousness. Rowland (2019), in her writing on the Kent coalfield, explored the relationship between body pedagogics and occupational health, drawing on Bourdieu's theoretical understanding of habitus (1984) to reveal how communal experiences shaped not only individual but collective attitudes towards embodiment and illness. Given that wider networks of support are providers of informal care (and therefore often at the forefront of the stroke survivors' battles with their changing body), it is important that we recognise the collective experiences of embodiment to understand both the challenges that they experience in accepting differences in their relative's behaviour and personality after stroke, and also the implications for their own lives and relationships:

When she had the stroke, yes, she was a completely different person, not physically, but sort of mentally … Whereas, and her personality, she was not herself at all, that everybody who knew about the stroke, the close family members, realised. She didn't remember anything at all. It was like she was in her own world. And no one around her. It was just recently, in the last couple of months where she has now, that she's herself. So, there was a period of time where she distanced towards me.

(Rebecca, daughter of Darianna)

Changes in a relative's behaviour and personality following a stroke were hard to overcome, particularly when as in this case, the daughter felt that it had diminished her relationship with her mother, as she appeared more ‘distanced towards [her]’. The stroke had altered her image of her mother, who now appeared ‘a completely different person’, changed not physically but mentally by the stroke, and she had at times felt disconnected from her mother. Another participant account provides further evidence of the impact that stroke can have in altering family relationships. Charlie chose to reflect on how the stroke had altered his father's personality, and subsequently, their relationship:

One of his charms was that he was very witty. There was always a bit of wit in everything that he said, and you’d struggle to keep up with him. That has very much diminished now. So, that has gone on the back burner. He needs to think quite a lot about what he’s saying before he says it. He often confuses words for other words. And if you’re not on his wavelength – fortunately, you know, I’ve been around him for a number of years, obviously, I know how he thinks. But he’s thinking sometimes before he does and what I have to do is stop myself – it’s, I suppose you could liken it to somebody that stutters, you want to finish the word for them. And when you do it, you think, ’Oops, I shouldn’t have done that.’ That’s where I find myself with my father, I don’t wish to do that, you know, I just let him finish what he wants to say. I’ve stopped correcting him as well on the words that he’s using, because it doesn’t help really.

(Charlie, son of Andrew)

Regret shaped the relationship the son had with his father. The perceived loss of his father's ‘wit’, which he described as ‘one of his charms’, following the stroke altered his image of his relative. The stroke had taken a part of his father's personality that he remembered fondly, and in doing so altered the dynamic of their relationship.

Stroke in diminishing bodily capabilities, leads to changes in the identities and character traits that individuals embody. For family, friends and carers, dealing with the aftermath of stroke could be equally challenging. Whilst these individuals did not directly experience the physical effects of stroke upon their body, it did have social ramifications, altering relationships partly in response to changes in the stroke survivor's personality.

## 4 Discussion

Drawing on Bury’s (1982) concept of biographical disruption, and particularly the three key forms, we utilise narratives of informal care to evoke theoretical discussion relating to embodiment in narrating experiences of stroke. Narratives of care, rehabilitation and social interactions emphasised the multiplicity of disruptions that stroke survivors and those around them experience. This has implications for a stroke survivor's identity and relationship with the social environment, with individuals appearing conscious of their body's capabilities.

These findings, in considering Engman's (2019) work alongside Cluley et al. (2023) within the context of stroke, add to ongoing sociological debates on the relationship between chronic illness and biographical disruption. For our participants, embodiment appears not as a ‘mechanism’, as described by Engman (2019), but an outward reflection of the reoccurring disruptive impact that their stroke has had upon their lives. As is evident in our findings, we extend Cluley et al. (2023) ‘biographical dialectics’ through the inclusion of body studies. Embodiment is crucial in understanding ongoing lived experience. Thus, through the creation of ‘embodied dialectics’, we gain understanding of stroke survivors and their wider social interactions. We argue that in examining ‘biographical dialectics’ in relation to embodiment in the context of care practices, which we term embodied dialectics, three distinct but interlinking aspects are revealed. Firstly, contradictions are generative: the distinction between past, present and future understanding of embodiment leads individuals to produce new forms of embodied knowledge.

Secondly, we see evidence of tensions that create motion, thus ensuring an ongoing dialectical process that generates creative adaptations and conversations in relation to informal care and embodied practices post‐stroke. Our participants, as stroke survivors, illustrate how their bodies ‘remember’ previous capabilities, and solutions generate new forms of embodiment. For instance, Richard's walking stick creates ‘moral support’ but also an embodied awareness of dependence that did not exist before his stroke. Edmund's clothes pegs transform speech therapy into shared and ongoing humour, while Joseph uses photographs to control his identity narrative, but also reinforce temporal embodied rupture. While asking about informal care practices, what appears are rich accounts of the lived experiences of stroke, which provide insights into individuals' ability and drive for independence, to limit the disruptive effects of their chronic illness. In performing tasks and engaging in social activities enjoyed and experienced prior to stroke, participants were made aware of their bodily ‘dys‐appearance’ (Leder 1990). Furthermore, whilst this paper did not choose to focus on the psychological impacts of stroke, conversations relating to levels of independence and care responsibilities highlight the need for further consideration of the role that mental health plays within the construct of embodiment post‐stroke and stroke research more broadly.

Lastly, there is no final synthesis—the body remains a site of productive tension. This is highlighted in the implications for care as we see evidence of the importance of autonomy to stroke survivors, whilst acknowledging the rupturing consequences of the condition. Much has been written on the contributions of carers and informal care practices in the lives of those individuals learning to live with chronic illness. As Quinn et al. (2014) and Bertilsson et al. (2014) illustrate, the physical and emotional implications of stroke are felt not only by the survivors but also by carers themselves, who are often at the forefront in experiencing the consequences of embodied changes. This is highlighted above through the cases of Rebecca and Charlie in coming to terms with changes in their parents' behaviour and personality. When considering the relationship between biographical disruption and embodiment in relation to the role of informal carers, Bury's concept may have a certain value in allowing us to understand the disruptive effects of stroke within the context of societal and lay understandings of informal care. For example, a study examining the relationship between frailty and biographical disruption recognised issues surrounding isolation from society and the need to mobilise resources to support changes in health (Cluley et al. 2021). Therefore, it is important that we acknowledge that different experiences of stroke exist not only in terms of physical bodily impact but within the wider social context of everyday interactions and care. Stroke is not only embodied on an individual level but is a collective experience that has implications for wider networks of support. Family, friends and carers, despite having no physical experience of stroke themselves, are often at the forefront of stroke survivors' social and cultural bodily experiences. Whilst ‘time spent’ or ‘ageing’ with a chronic condition did not make changes, complications or losses any easier (Larsson and Grassman 2012, 1166), individuals adapted to their body's capabilities. Stroke can have a major impact on what someone can do for their work and in their social life. Whilst they recognised their body had been permanently changed, they did not wish to be defined by their stroke. Therefore, collective embodiment provides an additional site for these tensions to be past, present and future ‘embodied dialectics’ to foster and materialise, with implications not just for individual embodiment but collective embodiment as experienced by their wider network.

This research examines informal care brought to the fore, embodiment and its relationship to stroke. This study's strength lies in its recognition of the connection between the physical impact of stroke, changes in social activities and collective embodiment. Moreover, in asking individuals to narrate their lived experiences of informal care, we found that this led individuals to develop an awareness of their body's capabilities post‐stroke and pre‐stroke. Participants reflected on the changes that their bodies had undergone, which were seen as negative and led to changes in individuals' perceptions of identity. However, a limitation of the study relates to its limited sample size, as although participants were drawn from a diverse population, it was not possible to consider how embodiment/experiences may differ with regard to demographic variations (geographical location, age, ethnicity, socio‐economic status and time since stroke). Moreover, this meant it was not possible to fully consider the implications that this had, particularly for communal/community aspects of informal care and management of life after stroke. We also recognise that time is an important factor in individuals' acceptance of their bodily changes, but further data collection is needed over an extended period to determine the impact that this has on stroke survivors' post‐stroke experience.

The relationship between biographical disruption and embodiment within the context of stroke raises questions as to how we might understand and interpret individuals' management of their health conditions and social relationships. Whereas disruption can be along numerous lines (impact of illness, life pre‐stroke etc.), the way we think about stroke care needs to consider the whole person. This includes giving individuals an opportunity to have a voice in the care and support that they receive, alongside more support for individuals looking after people experiencing chronic illness or disability, all issues that are key to the NHS Long Term Plan's focus on ageing well (NHS England 2019). Because strokes are occurring at younger ages and people are surviving longer, these considerations are even more pressing. Drawing on sociological theories relating to both embodiment and biographical disruption together and foregrounding not only the individual but the collective impacts of stroke on an ‘embodied dialectic’, this paper provides a path forward in taking seriously the varied and contextually dependent ways stroke can Disrupt individuals and those around them.

## Author Contributions

**Sophie Rowland‐Coomber:** conceptualization (equal), data curation (lead), formal analysis (lead), investigation (lead), methodology (supporting), project administration (lead), writing – original draft (lead), writing – review and editing (lead). **Eleanor Stevens:** writing – original draft (supporting). **Christopher McKevitt:** funding acquisition (equal), writing – original draft (supporting). **Nicholas J. Williamson:** formal analysis (supporting), investigation (supporting), writing – review and editing (supporting). **Iman Muzafar:** formal analysis (supporting), investigation (supporting), writing – review and editing (supporting). **Timothy Neate:** funding acquisition (equal), writing – review and editing (supporting). **Martin Chapman:** funding acquisition (equal), writing – review and editing (supporting). **Ajay Bhalla:** funding acquisition (equal), writing – review and editing (supporting). **Charles D. A. Wolfe:** funding acquisition (equal), writing – review and editing (supporting). **Iain J. Marshall:** funding acquisition (equal), writing – review and editing (supporting). **David Wyatt:** conceptualization (equal), data curation (supporting), formal analysis (equal), funding acquisition (equal), investigation (supporting), methodology (lead), project administration (supporting), supervision (lead), writing – original draft (supporting), writing – review and editing (supporting).

## Funding

This project is funded by the National Institute for Health and Care Research (NIHR) under its Programme Grants for Applied Research (NIHR202339) and is supported by the NIHR Applied Research Collaboration (ARC) South London at King's College Hospital NHS Foundation Trust. The views expressed are those of the authors and not necessarily those of the NIHR or the Department of Health and Social Care.

## Data Availability

The datasets generated and analysed during the study are not publicly available but may be available from the corresponding author on reasonable request.
